# Alcohol and survival in ESCC: Prediagnosis alcohol consumption and postoperative survival in lymph node-negative esophageal carcinoma patients

**DOI:** 10.18632/oncotarget.8754

**Published:** 2016-04-15

**Authors:** Qilong Ma, Wengao Liu, Ran Jia, Hao Long, Lanjun Zhang, Peng Lin, Hongyun Zhao, Guowei Ma

**Affiliations:** ^1^ Sun Yat-sen University Cancer Center, Guangdong Esophageal Cancer Institute, State Key Laboratory of Oncology in South China, Collaborative Innovation Center for Cancer Medicine, Guangzhou, China

**Keywords:** alcohol consumption, postoperative survival, esophageal carcinoma, Chinese cohort

## Abstract

**Background:**

The association between esophageal cancer and prediagnosis alcohol consumption is well established. However, evidence that prediagnosis alcohol consumption affects postoperative survival in patients with lymph node-negative esophageal squamous cell carcinoma (ESCC) is lacking. We conducted a retrospective study on the effect of prediagnosis alcohol consumption on the postoperative survival of patients with lymph node-negative ESCC in China.

**Methods:**

We enrolled 643 ESCC patients with negative lymphatic metastasis who had undergone esophagectomy between 1990 and 2005 at the Department of Thoracic Surgery, Sun Yat-sen University Cancer Center, Guangzhou, China, and reviewed their demographic, pathologic, preoperative, and cancer outcome data obtained from medical records. These data were analyzed using life table and Kaplan–Meier analyses and multivariate Cox regression.

**Results:**

There was a significant reduction in 3- and 5-year survival in drinkers with lymph node-negative ESCC. For drinkers, 3- and 5-year survival rates were 43% and 36% respectively, whereas, for nondrinkers, the corresponding values were 63% and 58%, respectively (*p* < 0.05). Multivariate Cox regression showed that drinking (*p* = 0.001, relative risk =1.583) was an independent factor for survival in patients with lymph node-negative ESCC. Striated analysis revealed that drinking was an independent factor for survival in patients with stage II A (*p* = 0.008, relative risk =1.679), stage IB (*p* = 0.044, relative risk=1.517), and well (*p*=0.011, relative risk =1.783) and moderately (*p* = 0.002, relative risk = 1.915) differentiated ESCC.

**Conclusions:**

Prediagnosis alcohol consumption is an independent prognostic factor for postoperative survival in patients with lymph node-negative ESCC.

## INTRODUCTION

Esophageal cancer is one of the most common malignant tumors, ranking sixth in the causes of cancer mortality worldwide [1]. It is prevalent in China, Iran, South Africa, Uruguay, France, and Italy, of which China has almost half the total cases and the highest mortality rate [2]. In China, esophageal cancer is primarily of the squamous cell type, which accounts for >95% cases [3]. Despite improvements in nonsurgical treatment, surgery remains the mainstay of curative treatments. The outcome of surgical resection for esophageal cancer is poor. Although the postoperative 5-year survival rate of esophageal cancer is just 20%-40% in China [4], it is still higher than that reported in Western countries [5].

Numerous studies on esophageal cancer etiology have been conducted over the past decade. Smoking and prediagnosis alcohol consumption are well-known risk factors [6–9]. Many factors have been identified as prognostic, including tumor biological behavior, postoperative treatment, operative technique, and response to preoperative chemoradiotherapy [10]. Alcohol consumption is rising worldwide, and, according to a recent World Cancer Research Fund report [11], is a convincing risk factor for esophageal cancer. Given the role of alcohol consumption in the etiology of esophageal cancer, it is reasonable to hypothesize that it also influences tumor progression and patient survival.

We report the findings of a large retrospective study in a cohort of patients with lymph node-negative esophageal squamous cell carcinoma (ESCC), where in the influence of prediagnosis alcohol consumption on postoperative survival in patients with different grades and stages of ESCC, as well as the mechanism underlying these effects was investigated.

## MATERIALS AND METHODS

We performed a large retrospective patient analysis by searching the esophageal cancer database of the Department of Thoracic Surgery at Sun Yat-sen University Cancer Center, Guangzhou, China. We enrolled 643 patients with negative lymphatic metastasis who had undergone esophagectomy between 1990 and 2005 at this institute. Patients were not eligible if tumors were located at the cervical esophagus or esophagogastric junction, or had other histological subtypes of esophageal cancer besides ESCC. None of the patients died as postoperative complication, and none received preoperative chemotherapy or irradiation. Sun Yat-sen University Cancer Center Hospital Ethics Committee approved the study. Mean follow-up duration was 6.5 years (range: 1-20 years).

Clinical and pathological data were extracted from medical records. Baseline data included age, sex, smoking, alcohol consumption, family history, surgical technique, and tumor biological features. Tumors were staged according to the American Joint Committee on Cancer (AJCC) Staging Manual (6^th^ Edition). Patients with a present or past history of alcohol consumption were referred to as drinkers.

**Figure 1:**
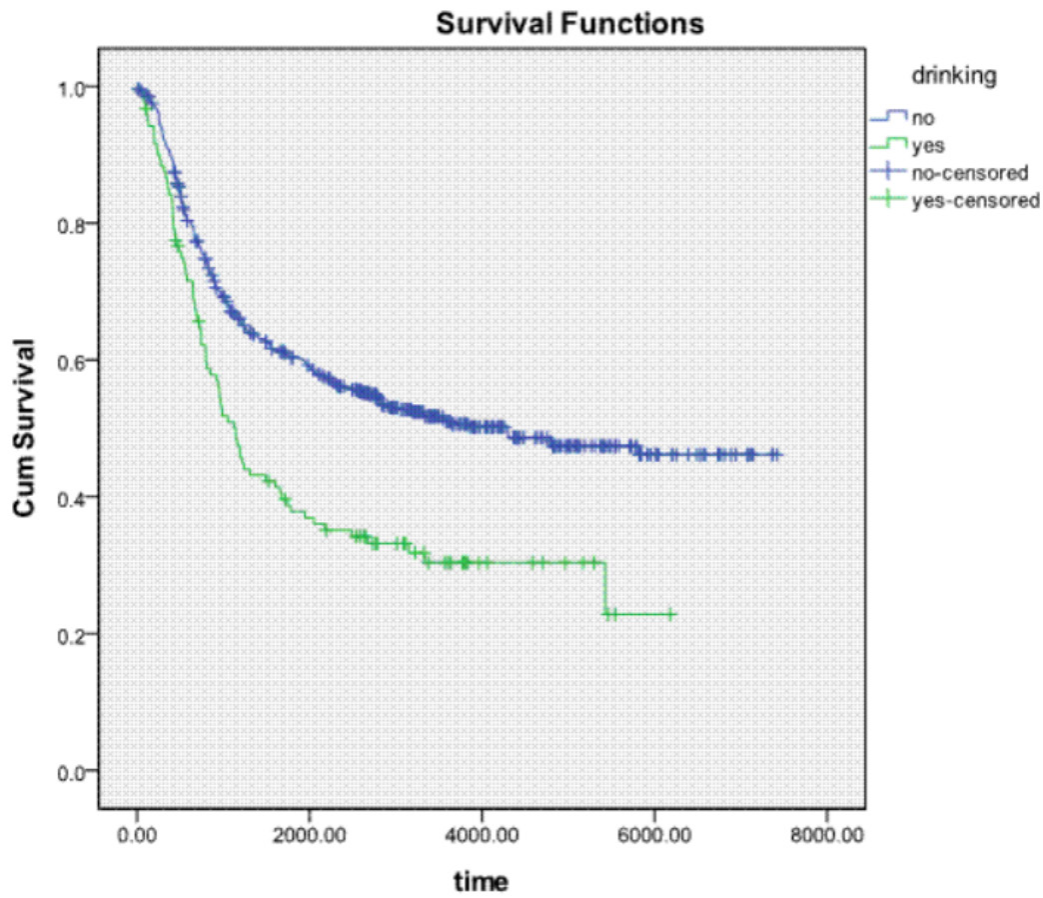
Kaplan-Meier survival curve of patients grouped by drinking and nondrinking habit

Analyses were conducted using SPSS version 18.0 statistical software (IBM SPSS, Inc., Chicago, IL, USA). Descriptive statistics (e.g. frequency, mean, standard deviation) were obtained for demographic, epidemiologic, and clinical patient characteristics. Life table analysis was used to calculate the 3- and 5-year survival rates of patients, whereas Kaplan-Meier analysis was used to calculate their survival probability. Survival curves were generated according to drinking history (Figure 1), and the log rank test was used to determine the statistical significance of differences between the survival curves of drinkers and those of nondrinkers. Stage-stratified analysis with the logrank test was used to study the influence of alcohol consumption on cancer at different stages. A *p*-value of <0.05 was considered statistically significant. Multivariate Cox regression was used to exclude other confounding factors affecting survival. Survival was defined as time from date of surgery to date of death, with living patients censored at the date of last follow-up or date of analysis.

## RESULTS

### Patient groups according to drinking history

Patients were categorized as drinkers or nondrinkers on the basis of their drinking history. Table 1 summarizes patient demographics. There were 121 (18.8%) drinkers and 522 (81.2%) nondrinkers. Drinkers were more commonly male (118; 97.5%; *P* < 0.05), and most of them had a smoking history as well (111; 91.7%; *P* < 0.05). Tumor location was significantly different (*p* = 0.006) between drinkers and nondrinkers. No significant differences in age, family history, tumor features, surgical technique, or postoperative treatment were evident between the two groups.

**Table 1:** Baseline characteristic grouped by history of drinking

	drinker	non-drinker	*P*-value
gender			<0.05
Male	118(97.5%)	336(64.4%)	
Female	3(2.5%)	186(35.6%)	
age			0.275
<40	3(2.5%)	30(5.7%)	
40~60	80(66.1%)	318(60.9%)	
>60	38(31.4%)	174(33.3%)	
Smoker	111(91.7%)	278(53.3%)	<0.05
family history	8(6.6%)	38(7.3%)	0.797
Tumor location			0.006
upper thoracic	17(14%)	54(10.3%)	
middle thoracic	73(60.3%)	389(74.5%)	
lower thoracic	31(25.6%)	79(15.1%)	
post-operative stage			0.777
IA	8(6.6%)	40(7.7%)	
IB	55(45.5%)	249(47.7%)	
IIA	58(47.9%)	233(44.6%)	
tumor grade			0.229
well	43(35.5%)	198(37.9%)	
moderately	58(47.9%)	209(40.1%)	
poorly	20(16.5%)	114(22%)	
surgery incision			0.628
right thoracic	26(21.5%)	118(22.6%)	
Left thoracic	95(78.5%)	404(77.4%)	

*1 value of tumor grade is missing.

According to the results of life table analysis, the 3- and 5-year survival rates were 43% and 36%, respectively, in drinkers and 63% and 58%, respectively, in nondrinkers. According to the results of Kaplan-Meier analysis and the logrank test, overall survival duration was significantly longer in nondrinkers than in drinkers (*p* < 0.05).

The results of multivariate Cox regression (Table 2) indicated that drinking [relative risk (RR) = 1.583, *p* = 0.001], surgical technique (RR = 1.107, *p* = 0.023), postoperative staging (RR=1.332, *P* = 0.002), and tumor grade (RR = 1.182, *p* = 0.027) were independent prognostic factors for survival in patients with ESCC.

**Table 2:** Multivariable analysis of factors related to ESC survival

			95.0% CI for Exp(B)	
	*P*	HR	Lower	Upper
gender	.422	.859	.592	1.245
Age	.887	1.015	.830	1.241
smoking	.153	1.280	.913	1.794
drinking	.001	1.583	1.213	2.074
Family history	.388	.814	.511	1.298
Tumor location	.703	.958	.770	1.193
Surgery technique	.023	1.107	1.014	1.209
Post-operative staging	.002	1.332	1.108	1.602
Tumor grade	.027	1.182	1.019	1.372

### Drinking and postoperative staging

**Figure 2:**
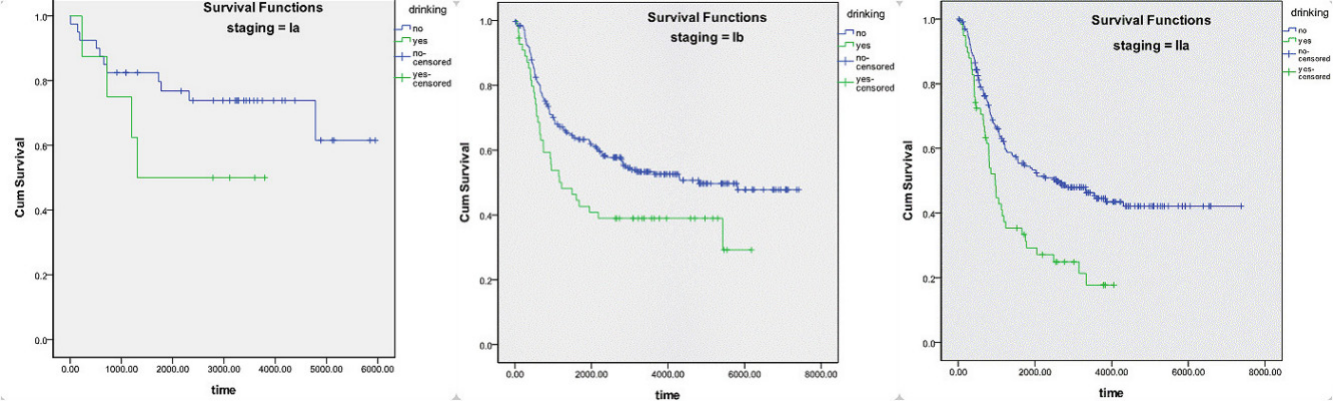
Kaplan-Meier survival curve striated by postoperative staging of ESCC

The results of stage-stratified analysis are demonstrated in Figure 2 and Table 3. Results of the logrank test indicated that drinking had a significant influence on survival in patients with stage IB (*p* = 0.015) and stage IIA (*p* < 0.01) ESCC, with no effect in patients with stage IA (*p* = 0.190) ESCC. In addition, multivariate analysis (Table 3), which excluded confounding factors, showed that drinking was an independent factor for survival in patients with Stage IIA (*p* = 0.008; RR = 1.679) and stage IB (*p* = 0.044; RR = 1.517) ESCC, with no effect in patients with Stage IA ESCC.

**Table 3:** Multivariable analysis of factors related to ESC survival grouped by staging

	IA		IB		IIA	
	*P*	RR	*P*	RR	*P*	RR
gender	0.644	0.567	0.369	1.285	0.024	0.532
age	0.089	0.376	0.786	1.041	0.398	1.138
smoking	0.316	3.335	0.126	1.480	0.819	0.946
**drinking**	0.084	4.224	0.044	1.517	0.008	1.679
Family history	0.077	0.104	0.199	0.624	0.791	1.092
location	0.082	0.247	0.704	0.942	0.484	1.126
surgery	0.140	1.482	0.113	1.111	0.215	1.086
Differentiation grade	0.021	3.206	0.035	1.266	0.698	1.044

### Drinking and differential grade

**Figure 3:**
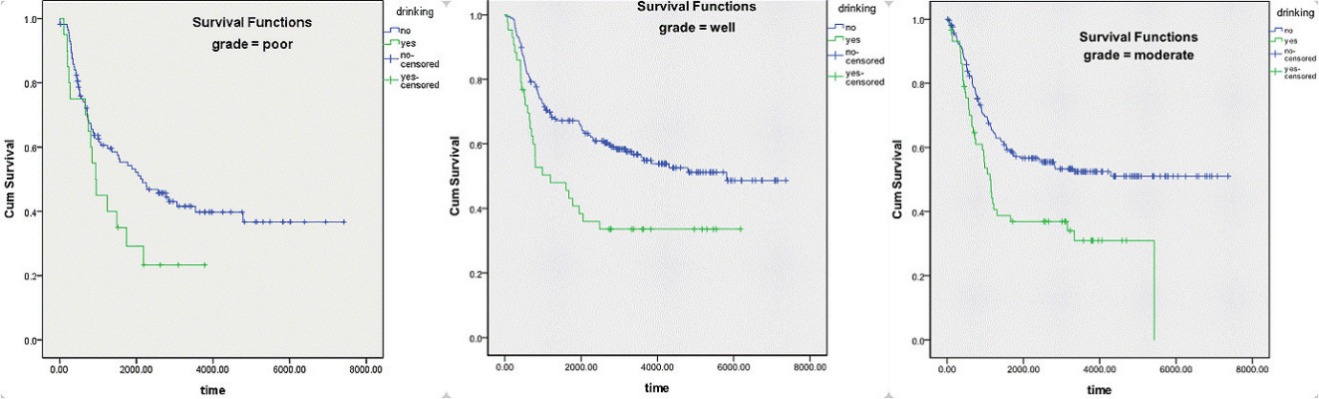
Kaplan-Meier survival curve striated by grade of differentiation of ESCC

Figure 3 and Table 4 show results of survival analysis stratified by differential grade. Results of the logrank test indicated that drinking had a significant influence on survival in patients with well (*p* = 0.003) and moderately differentiated (*p* = 0.002) ESCC, with no effect in patients with poorly differentiated ESCC (*p* = 0.65). In addition, multivariate analysis (Table 4), which excluded confounding factors, showed that drinking was an independent factor for survival in patients with well (*P* = 0.011, RR = 1.783) and moderately differentiated ESCC (*P* = 0.002, RR = 1.915), with no effect in patients with poorly differentiated ESCC

**Table 4:** Multivariable analysis of factors related to ESC survival grouped by differentiation grade

	well		moderate		poor	
	*P*	RR	*P*	RR	*P*	RR
gender	0.789	1.092	0.262	0.726	0.573	0.781
age	0.999	1.000	0.857	0.971	0.620	1.108
smoking	0.018	1.959	0.404	0.797	0.456	1.349
drinking	0.011	1.783	0.002	1.915	0.390	1.308
Family history	0.783	1.116	0.460	0.773	0.266	0.508
location	0.303	0.821	0.217	1.254	0.222	0.754
surgery	0.398	1.066	0.010	1.206	0.697	1.038
Post-operative staging	0.014	1.486	0.025	1.408	0.922	1.018

## CONCLUSIONS

Esophageal cancer is currently the fifth most common and fourth most lethal malignant tumor in China. Although the incidence of esophageal adenocarcinoma is rising in Western countries, it remains unchanged in China, where ESCC accounts for the majority of esophageal cancer cases [3, 19].

Numerous studies have been conducted to determine the prognostic factors for ESCC survival. Various biological aspects of tumors reportedly correlate with prognosis. Tumor grade and stage are well-known prognostic factors, especially high tumor grade and stage [12–14]. In our study, multivariate analysis consistently identified postoperative staging (*P* = 0.002, RR=1.332) and tumor grade (*p* = 0.027, RR = 1.182) as factors influencing survival, further confirming the role of biological factors in the survival of patients with ESCC.

Pathological information is valuable for predicting prognosis in esophageal cancer patients. However, this is not accurate enough, as prognosis can also be influenced by prediagnostic and postoperative factors. Our study focused on the influence of drinking on ESCC survival and demonstrated that drinking significantly reduces the 3- and 5-year survival rates of patients with ESCC. Multivariate Cox regression also indicates drinking as an independent factor influencing survival in these patients. Potential mechanisms by which alcohol consumption may affect survival include increase in local permeability [15], suppression of immune function [16], and generation of metabolites that are carcinogenic to humans [17].

Stage-stratified analysis demonstrated a significant difference in survival between drinkers and nondrinkers with stage IIA or stage IB ESCC. According to analysis striated by grading, a significant difference in survival was observed between drinkers and nondrinkers with well or moderately differentiated ESCC. Possible explanations are described below. Firstly, there lack of power in the stratified datasets. There were only 8 drinkers and 40 nondrinkers with stage IA ESCC; these numbers were far from enough to display significance. The situation was similar with poorly differentiated ESCC, which was identified in 20 drinkers and 114 nondrinkers. As Figures 2 and 3 show, there is a trend of curve separation for Stage IA and poorly differentiated ESCC. The second possible explanation could be the different impact of drinking on tumors of different grades and stages. It is known that alcohol dehydrogenase (ADH) and aldehyde dehydrogenase play a key role in ethanol metabolism. Total ADH activity is significantly higher in cancer tissues than in healthy organs [18]. We conclude that our results can be explained by variable ADH activity in different grades and stages, leading to an enhanced accumulation of toxic acetaldehyde.

The strengths and limitations of our study should be considered while interpreting these results. Our strengths include a large sample of consecutive patients from a well-maintained database and an efficient recording medical system containing abundant tumor information, such as tumor grade and stage. Moreover, our study showed that alcohol consumption prior to diagnosis, a well-known risk factor for cancer development, also appears to affect cancer outcome. This suggests that any drinking is a risk factor for ESCC patient survival.

Our study has all the constraints of retrospective analysis. First, a comparison of drinkers and nondrinkers is subject to selection bias. Many studies have shown that nutrition, diet, and socioeconomic factors also affect the survival of ESCC patients [20–22]. However, we failed to investigate these factors, which may have had an influence on survival in our study.

In our study, we staged tumors according to the AJCC Staging Manual (6^th^ Edition) because it was a retrospective study that enrolled patients between 1990 and 2005. According to the sixth edition, tumors should be sectioned according to tumor center location, with the carina as a reference point. Conversely, according to the new edition, tumors should be sectioned according to their upper border, with the azygos vein and the inferior pulmonary vein as reference points. Therefore, because retrospective use of the latest edition was difficult and could lead to inaccuracy, we used the sixth edition of the AJCC Staging Manual in our study.

Despite these limitations, our study identified prediagnosis alcohol consumption as an independent factor for postoperative survival in patients with lymph node-negative ESCC after adjusting for other confounding factors, such as sex, smoking and tumor features. Decreasing the daily intake of alcohol is therefore important for preventing ESCC development or for obtaining a good prognosis in patients with ESCC.
